# Synergistic Effect of Anethole and Platinum Drug Cisplatin against Oral Cancer Cell Growth and Migration by Inhibiting MAPKase, Beta-Catenin, and NF-κB Pathways

**DOI:** 10.3390/ph16050700

**Published:** 2023-05-05

**Authors:** Abdelhabib Semlali, Ikram Ajala, Sarra Beji, Mohammed Mousa Al-Zharani, Mahmoud Rouabhia

**Affiliations:** 1 Groupe de Recherche en Écologie Buccale, Faculté de Médecine Dentaire, Université Laval, Quebec, QC G1V0A6, Canada; 2 Biology Department, College of Science, Imam Mohammad Ibn Saud Islamic University (IMSIU), Riyadh 11623, Saudi Arabia

**Keywords:** oral cancer, chemotherapy, cisplatin, anethole, proliferation, apoptosis

## Abstract

Cisplatin is a common drug used to treat patients with oral squamous cell carcinoma. However, cisplatin-induced chemoresistance poses a major challenge to its clinical application. Our recent study has shown that anethole possesses an anti-oral cancer effect. In this study, we examined the combined effect of anethole and cisplatin on oral cancer therapy. Gingival cancer cells Ca9-22 were cultured in the presence of various concentrations of cisplatin with or without anethole. The cell viability/proliferation and cytotoxicity were evaluated, respectively, by MTT, Hoechst staining, and LDH assay, while colony formation was measured by crystal violet. Oral cancer cell migration was evaluated by the scratch method. Apoptosis, caspase activity, oxidative stress, MitoSOX, and mitochondrial membrane potential (ΔΨm) levels were evaluated by flow cytometry, and the inhibition of signaling pathways was investigated by Western blot. Our results show that anethole (3 µM) potentiates cisplatin-induced inhibition of cell proliferation and decreases the ΔΨm on Ca9-22 cells. Furthermore, drug combination was found to inhibit cell migration and enhanced cisplatin cytotoxicity. The combination of anethole and cisplatin potentiates cisplatin-induced oral cancer cell apoptosis through the activation of caspase, while we also found anethole and cisplatin to enhance the cisplatin-induced generation of reactive oxygen species (ROS) and mitochondrial stress. In addition, major cancer signaling pathways were inhibited by the combination of anethole and cisplatin such as MAPKase, beta-catenin, and NF-κB pathways. This study reports that the combination of anethole and cisplatin might provide a beneficial effect in enhancing the cisplatin cancer cell-killing effect, thus lowering the associated side effects.

## 1. Introduction

Oral cancer, a type of head and neck cancer, is ranked as the sixth most prevalent cancer globally [1]. The disease is multifaceted and influenced by various risk factors such as alcohol consumption, tobacco use, and poor dietary habits. Roughly 7–19% of oral cancer cases can be attributed to heavy alcohol intake, and 10–15% are due to inadequate fruit and vegetable consumption. Human papillomavirus (HPV), particularly types 16 and 18, are high-risk factors for oral cancer, particularly in individuals who do not smoke or consume alcohol [2,3,4]. Oral cancer treatment often involves the use of surgery in combination with radiotherapy or chemotherapy [5]. The current gold standard of therapy involves the combination of chemotherapy such as cisplatin and radiotherapy. Resistance to platinum-based chemotherapy is a major setback in cancer therapy. The most common chemotherapeutic drug used is cisplatin [6]. The biological mechanism of cisplatin involves the induction of DNA damage which in turn causes the inhibition of cell proliferation and apoptosis of cancer cells [7]. Irrespective of its success as an anticancer drug, cisplatin application in cancer treatment is limited because of the acquired or intrinsic resistance leading to relapse and therapeutic failure, subsequently making some oral tumors more resistant to chemotherapy [8]. To overcome cisplatin resistance and reduce its side effects, it is necessary to develop an alternative chemotherapeutic or complementary strategy to treat oral cancer. One of the promising solutions is the use of natural products or their derivatives in combination with cisplatin. Recently, herbal-based constituents have drawn increasing attention due to their potential in enhancing the chemosensitivity of cancer cells to cisplatin and due to their biological activities, easy availability, and lack of toxic side effects [9]. Hence, plants and their bioactive compounds can be explored as safer alternatives in the form of combinational treatment strategies in addition to cisplatin. The recognition of plant products as effective and inexpensive sources of synthetic novel chemotherapeutic compounds is increasing. Therefore, research into anticancer supplements having the potential to kill malignant cells and decrease cytotoxicity in normal cells is of great importance in current anticancer therapies [10]. Furthermore, natural product has been heavily investigated in recent years [11]. Among these natural products, flavored compounds such as Curcuma, and quercetin have been shown and validated for their anticancer activity [12,13]. Plants are a vast source of nutraceuticals with antioxidant and anti-inflammatory properties, including carotenoids such as β-carotene, α-carotene, and lycopene; and polyphenols such as flavones, flavanols, isoflavones, catechins, curcumin, and resveratrol [14]. The anticancer activity of these compounds has been widely demonstrated in different colon cancer cell lines [13]. Furthermore, curcumin has been shown to possess synergistic effects with antineoplastics such as 5-fluorouracil and oxaliplatin, as well as an anti-inflammatory drug by inhibiting cyclooxygenase-2 and the NF-κB in colorectal cancer [12]. As in the above compounds, a natural product called anethole, a flavored and major compound extracted from anise, fennel, and licorice, may possess anticancer properties [15,16,17]. Anethole is a translucent, colorless amber liquid. By dint of its sweet flavor, it is used as a sweetener and in many alcoholic drinks. It is slightly soluble in water but greatly soluble in alcohol and it is also miscible with ether and chloroform [18]. It has been reported that bioactive components of anethole possess antioxidant, anti-inflammatory, chemo-preventive, antifungal, antiviral, and antibacterial activities [18,19,20]. Our previous study showed that anethole has a selective anticancer effect on oral cancer cells by triggering apoptosis, autophagy, and oxidative stress and by modulating MAPkases, NF-κB, and Wnt pathways [21]. Furthermore, Shahbazian et al. have reported the cytotoxicity of trans-anethole on two breast cancer cell lines, MCF-7 and T47D, using pelagic liposomal trans-anethole nanoparticles [22]. Choo et al. reported that the anethole inhibited cell adhesion and invasion to the matrix in a concentration-dependent manner [23]. All these data suggest that anethole could be a safe molecule to treat many diseases including cancer [18,19,20]. The current study aims to assess the potential beneficial effect of the combined treatment of the low concentration of cisplatin and anethole in oral cancer therapy. We hypothesized that anethole will potentiate the anticancer effect of cisplatin by inhibiting cell proliferation, promoting programmed cell death, reducing oxidative stress, and modulating the signaling pathways associated with the development of oral cancer. By reducing cisplatin side effects, we will contribute to improvement in the quality of life of oral cancer patients and unravel a new treatment strategy that is more effective with fewer or no side effects.

## 2. Results

### 2.1. Synergistic Effect of Anethole and Cisplatin on Inhibition of Oral Cancer Cell Viability

**Figure 1:**
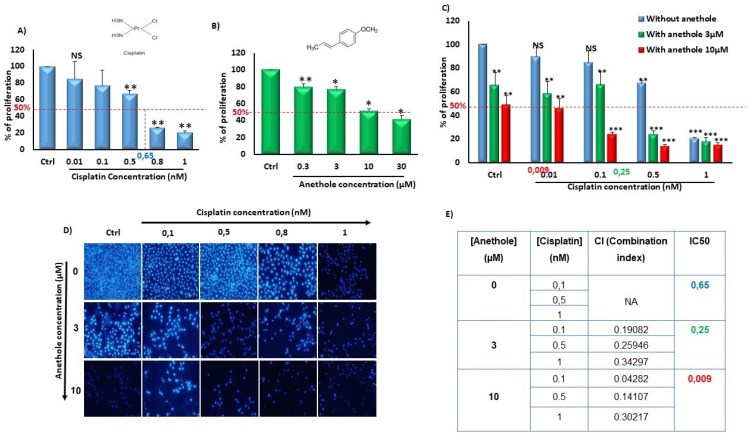
Synergistic effect of anethole and cisplatin on inhibition of oral cancer cell proliferation. (**A**) Anethole inhibits percent of cancer cell proliferation performed by MTT assay.NS: Non significant, * *p* < 0.05, ** *p* < 0.005, and *** *p* < 0.0005. (**B**) Anethole inhibits percent of Ca9-22 cell proliferation evaluated by MTT assay. (**C**) Effect of the combination of anethole with cisplatin on Ca9-22 cell proliferation. (**D**) Hoechst staining. (**E**) Table for combination index.

To evaluate the synergistic effect of anethole with cisplatin, we independently measured the effect of anethole and cisplatin on Ca9-22 proliferation followed by quantitating their combinational effect. Firstly, we have previously demonstrated the cytotoxic selected effect of anethole on oral cancer cells but not in non-tumorigenic gingival epithelial cells and fibroblast cells at low concentrations [21]. Figure 1 shows that anethole alone or in combination with cisplatin decreased oral cancer cell proliferation. In addition, the effect of cisplatin was concentration dependent on the inhibition of Ca9-22 proliferation with an IC_50_ containing 0.65 nM of cisplatin. In addition, there was a concentration-dependent decrease in oral cancer cell growth with cisplatin from 100% in untreated cells to 84.92 ± 8.20464% (*p* = 0.1), 77.07 ± 21.27% (*p* = 0.02), 66.86 ± 4.21% (*p* = 2.09 × 10^−6^), 26.25 ± 0.07% (*p* = 7.27 × 10^−19^), and 20.46 ± 2.12% (*p* = 1.89 × 10^−10^) with 0.01, 0.1, 0.5, 0.8, and 1 nM, respectively (Figure 1A). Anethole also exhibited an inhibitory effect on Ca9-22 proliferation with an IC_50_ of 10 µM at 24 h. A concentration-dependent decrease in cell growth was also observed for Anethole (Figure 1B). Combinational exposure to cisplatin and anethole induced a higher level of growth inhibition in Ca9-22 cells compared to a single exposure to each drug. The growth inhibition decreased from 100% in the control cells to 89.66 ± 9.5% (*p* = 0.15), 84.62 ± 10.33% (*p* = 0,08), 67.26 ± 1.6% (*p* = 2.49 × 10^−8^), and 20.59 ± 0.83% (*p* = 8.12 × 10^−14^) with, respectively, 0, 0.01, 0.1, 0.5, and 1 µM of cisplatin to 65.39 ± 11.01% (*p* = 0.01), 58.14 ± 10.53% (*p* = 0.002), 65.67 ± 10.85% (*p* = 0.006), 24.17 ± 2.67% (1.3 × 10^−9^), and 18.13 ± 3.16% (2.68 × 10^−12^) with the same concentrations of cisplatin combined with 3 µM of anethole. This decrease in cell proliferation induced by cisplatin was more enhanced when 10 µM of anethole was combined with cisplatin showing the lowest cell viability and highest cell viability at 48.68 ± 7.89% (*p* = 9.39 × 10^−5^), compared to cells exposed to cisplatin alone (Figure 1C). This result was also confirmed by the Hoechst staining which showed a decrease in the number of Ca9-22-stained nuclei with a single exposure to either anethole or cisplatin and combining any combination of the two drugs where there was a further reduction in the number of stained nuclei (Figure 1D). Our data showed that the combined anethole with different concentrations of cisplatin potentiates the cisplatin-induced inhibition of cell growth. It was observed that the IC_50_ decreased, respectively, from 0.65 µM for cisplatin alone to 0.25 µM for co-exposure to cisplatin and 3 µM of anethole to 0.009 µM with co-exposure to cisplatin and 10 µM of anethole. The CI (complementary index) values in Figure 1E of all the combination treatments were less than 1, suggesting there was a synergetic effect with a combination of cisplatin and anethole treatment.

**Figure 2:**
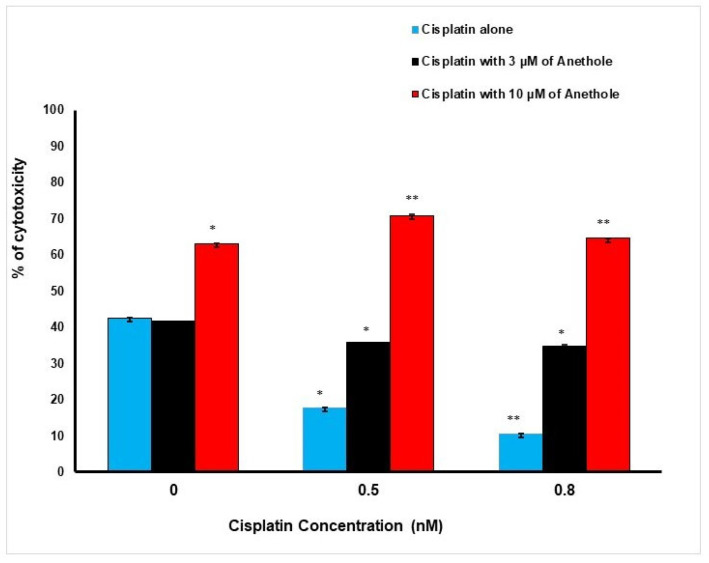
Synergistic effect of anethole and cisplatin on inhibition of oral cancer cell toxicity. LDH assay was measured after 24 h of treatment by anethole alone or by its combination with different concentrations of cisplatin. * *p* < 0.05 and** *p* < 0.005.

These findings are supportive of the LDH data. Inversely to proliferation, a higher concentration of anethole induced a higher cytotoxic effect on the cancer cells. The percentage of cell cytotoxicity was enhanced when cisplatin and anethole were combined. LDH activity assay demonstrated an increase in cytotoxic effect from 42.50 ± 0.03%, 17 ± 0.01%, and 10.6 ± 0.001% in cells treated, respectively, with 0, 0.5, and 0.8 nM of cisplatin to 41.47 ± 0.04%, 35.79 ± 0.04% and 34.86 ± 0.17%, when exposed to a combination of 3 µM of anethole to the respective concentration of cisplatin. This increase in toxicity was more pronounced when 10 µM of anethole was combined with different concentrations of cisplatin achieving 63.12 ± 0.005%, 70.80 ± 0.38%, and 64.56 ± 0.08% cytotoxicity when we combined 10 µM of anethole with cisplatin (Figure 2).

### 2.2. The Combination of Anethole and Cisplatin Potentiates the Inhibition of Colony Formation in Oral Cancer Cells

**Figure 3:**
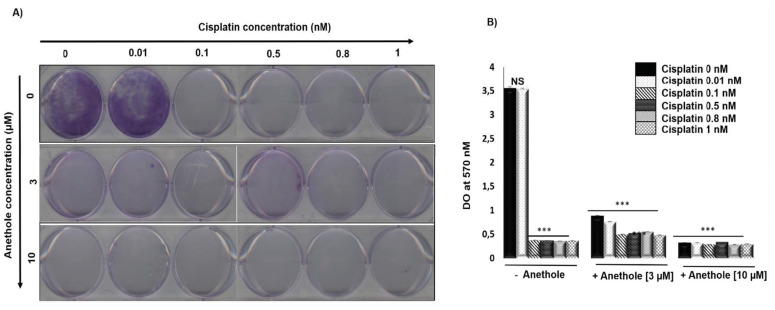
Anethole potentiates the effect of cisplatin inhibitor on colony formation in ca9-22 cells. (**A**) Colony formation through crystal staining. (**B**) Quantification of the number of colonies, the results were measured by Do at 570 nM after lysis by glacial acetic acid. *** *p* < 0.0005.

We investigated the effect of a combination of cisplatin and anethole on the inhibition of the colony formation in oral cancer cells. Figure 3A shows the representative images and Figure 3B shows the quantitative analysis of colony numbers where the data are presented as a percentage relative to the control cells. The results showed that cisplatin alone inhibited Ca9-22 colony formation from 0.01 nM. The combination of cisplatin with anethole had a higher inhibitory effect on colony formation and from 3 µM of anethole, we observed a complete inhibition of colony formation (Figure 3A). This data agreed with the crystal violet assay shown in Figure 3B. The combined treatment of cisplatin and anethole could inhibit the formation of colony formation better than either drug alone, which was consistent with the results from the viability assays in Section 1.

### 2.3. Anethole Potentiates Cisplatin on Inhibition of Cell Migration in Oral Cancer Cells

**Figure 4:**
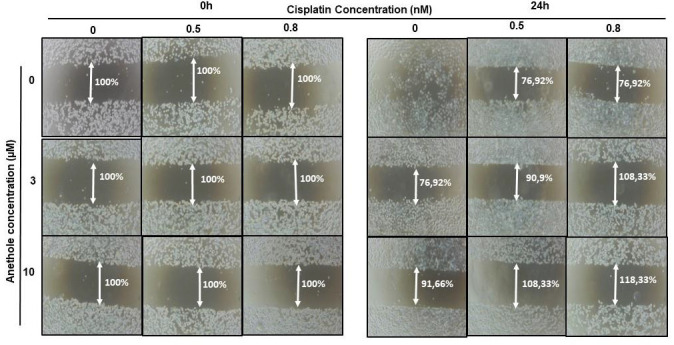
Anethole combined with cisplatin potentiates further oral cancer cell migration: Ca9-22 cells at 100% of confluence were scratched by 200 µL tips and then treated by anethole or cisplatin or with a combination of anethole and cisplatin. The percentage of the scratched area was measured after 24 h of treatment.

Migration/invasion is a hallmark of cancer cell characteristics required for metastasis. Thus, the treatment of metastatic cancer is often aimed at inhibiting cancer cell migration. Here, we investigated the effect of cisplatin alone or in combination with anethole on Ca9-22 migration using the scratch assay. As shown in Figure 4, anethole or cisplatin alone inhibited the migration of oral cancer cells in a concentration-dependent manner. However, this repression is enhanced when the cells were co-exposed to cisplatin and anethole. Furthermore, the scratch was completely closed at 24 h in the untreated cells. However, a combination of 0.5 nM of cisplatin and 3 µM of anethole inhibited cell migration as the size of the scratch was around 90.9%, and for a combination of 0.5 nM of cisplatin and 10 µM of anethole, the size was 108.33% at 24 h. To investigate the effect of combined cisplatin with anethole on epithelial-to-mesenchymal gene expression, we evaluated the expression of E-cadherin by Western blotting after treatment with a single drug and by a combination of two drugs (cisplatin and anethole). As shown by western blotting analysis, the expression of E-cadherin decreases considerably when the cells were treated with a combination of the two drugs. These suggest that anethole potentiates the effect of cisplatin against oral cell migration by blocking the epithelial-to-mesenchymal transition.

### 2.4. Anethole Synergy with Cisplatin in the Induction of Apoptosis via Caspase Activation

**Figure 5:**
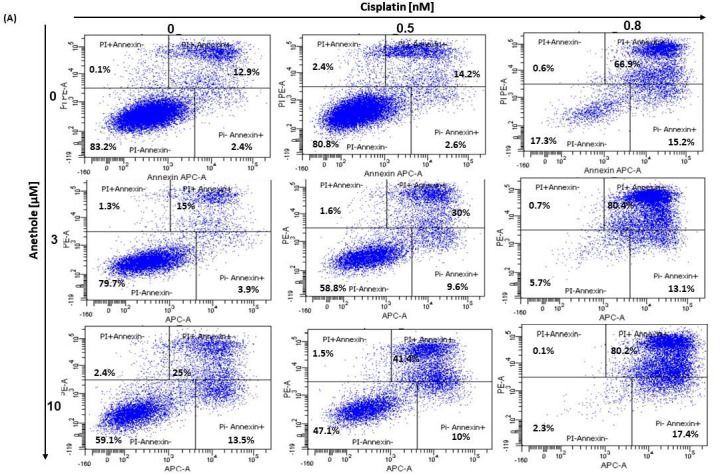

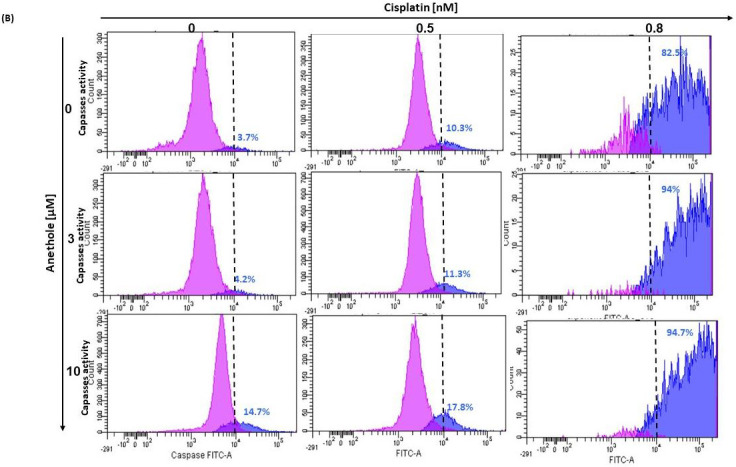
(**A**) Anethole combined with cisplatin potentiates further oral cancer cell apoptosis: Ca9-22 cells were treated by anethole or cisplatin or with a combination of these two drugs for 24 h. Apoptosis cells were evaluated by a PI/annexin kit using flow cytometry. (**B**) Anethole potentiates the effect of cisplatin on caspase activation; caspase activity was assessed using a caspase detection kit (TITC-VAD-FMK) and analyzed by flow cytometry using the FL1 channel.

The balance between apoptosis and proliferation is one of the determining factors of cancer development. As such, we investigated if there is a synergistic effect of anethole and cisplatin administration in oral cancer cell apoptosis. The apoptotic rate of oral cancer cells treated for 24 h with the different concentrations of cisplatin alone or in combination with 3 µM and 10 µM of anethole was determined by flow cytometry using annexin V-FITC/PI double staining. As shown in the Figure 5A, the percentage of live cells decreased from 83.2 ± 1.34% to 17.8 ± 10.11% upon treatment with cisplatin alone and to 59.1 ± 5.3% when the cells were treated with 10 µM of anethole. The combination of cisplatin at 0.5 nM with 3 µM of anethole decreases the living cells from 80.8% with cisplatin alone to 58.9% and to 47.5% when the cells were exposed to a combination of 0.5 nM of cisplatin and 10 µM of anethole. However, the percentage of apoptotic cells increased from 19.2% with 0.5 nM of cisplatin to 41.2% and 52.9%, respectively, when the cells were exposed to 0.5 nM of cisplatin and 3 µM or 10 µM of anethole (Figure 5A).

In addition, as shown in Figure 5B, anethole potentiates cisplatin-induced caspase activation in Ca9-22 cells. The percentage of Ca9-22 cells stained by FITC-CAD-FMK increases from 3.7% in untreated cells to 82.5% in cells treated with 0.8 nM of cisplatin and 14.7% in cells exposed to 10 µM of anethole. The combination of cisplatin with anethole induced more caspase activity to reach 94% when the cells were treated with the combination of 0.8 nM of cisplatin and 3 µM of anethole (Figure 5B). These suggest that anethole potentiates the apoptotic effect of cisplatin via the activation of caspase activity.

### 2.5. Anethole Potentiates Oxidative Stress Induced by Cisplatin

**Figure 6:**
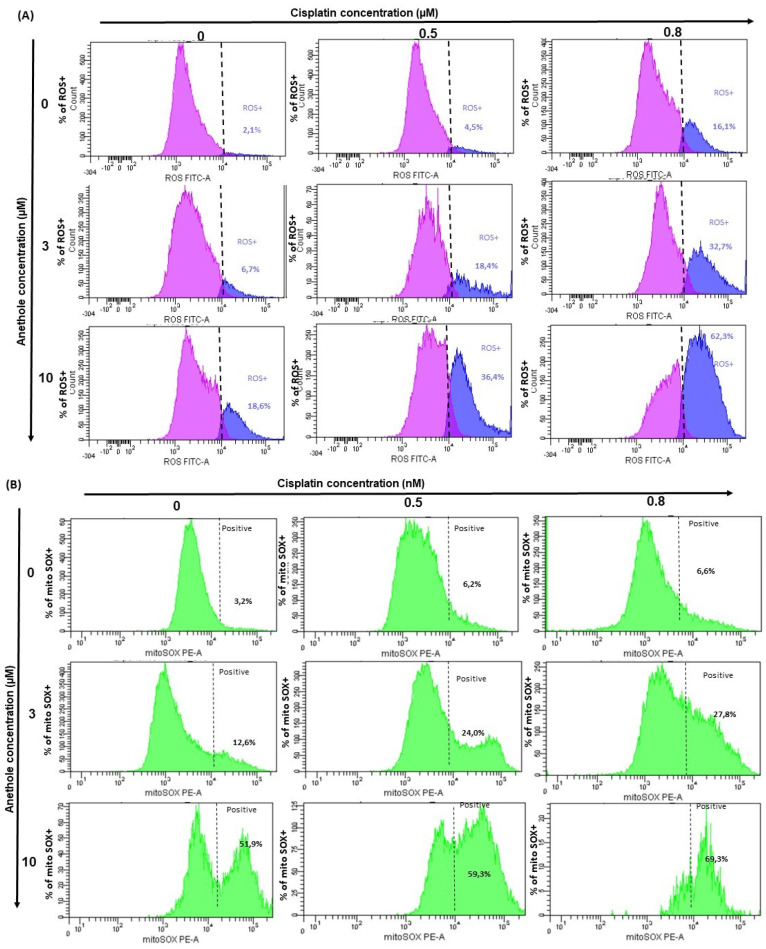
Anethole combined with cisplatin further potentializes the effect of cisplatin on the induction of oxidative stress in oral cancer cells. (**A**) The quantification of the percent of ROS-positive cells was performed by flow cytometry. The pink peak represents the percent of ROS-positive cells and the blue peak is the percentage of ROS-negative cells. (**B**) Effect of the combination of anethole and cisplatin on the induction of mitochondrial oxidative stress in Ca9-22 cells. MitoSox expression was performed by flow cytometry.

ROS are important regulators in various pathways including apoptosis and autophagy. To investigate whether treatment with anethole and cisplatin induces ROS generation in Ca9-22, oxidative stress was assessed by flow cytometry using two markers for reactive oxygen species (ROS). Initially, flow cytometry data showed the inhibition of ROS generation in the Ca9-22 cells treated with anethole and cisplatin for 24 h (Figure 6A). We found that this inhibition was more significant by combining the two drugs. Indeed, combining 0.5 µM of cisplatin and 10 µM of anethole significantly decreases the rate of intracellular ROS from 88.2% to 0.1%. However, the same result was only achieved for cisplatin alone by increasing its concentration 5-fold.

Then, we investigated the synergistic effect of anethole with cisplatin on mitochondrial stress. We evaluated MitoSOX production after treatment by a single drug and by a combination of two drugs. Figure 6B shows that the MitoSOX (+) (%) is dramatically increased with the co-treatment of anethole (10 µM) and cisplatin (0.8 µM). In addition, the percentage of MitoSOX (+) increased from 3.2% in the untreated cells to 69.3% for the cells exposed to a combination of 0.8 nM of cisplatin and 10 µM of anethole. These data suggest that anethole potentiates mitochondrial stress induced by cisplatin likely through the redox imbalance in the cell.

### 2.6. Anethole Synergy with Cisplatin in the Induction of Mitochondrial Membrane Potential (ΔΨm)

**Figure 7:**
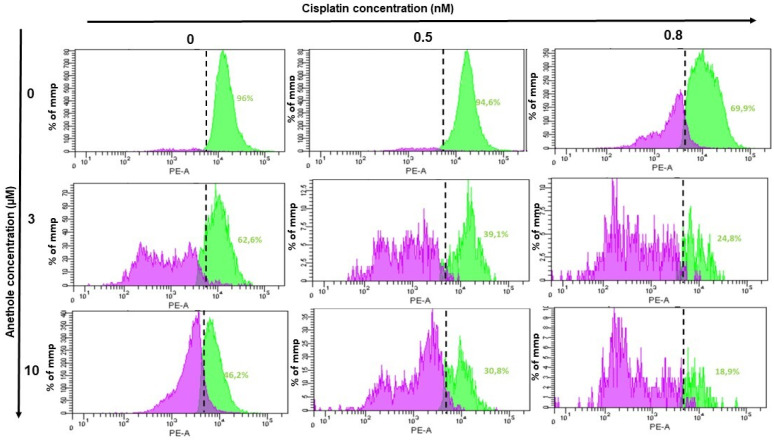
Effect of the combination of anethole and cisplatin on induction of mitochondrial membrane potential (ΔΨm) in Ca9-22 cells. ΔΨm expression was measured by flow cytometry.

Mitochondria membrane potential (ΔΨm) is a principal indicator of mitochondria activity. In this current study, we investigated whether combined anethole and cisplatin induce the cytotoxicity of oral cancer by the modulation of ΔΨm. Treatment of Ca9-22 cells with either cisplatin or anethole alone or a combination of these two drugs for 24h showed a reduction in ΔΨm from 96% in untreated cells to 69.9% for 0.8 nM cisplatin-treated Ca9-22 cells. The combination of 0.8 nM of cisplatin with 3 µM or 10 µM of anethole caused a concentration-dependent reduction in ΔΨm to 248% and 18.9%, respectively (Figure 7). These results suggest that the combination of cisplatin and anethole induced a reduction in membrane potential which would have resulted in reduced membrane integrity and the release of intracellular components, and subsequently, apoptosis, as we have shown above.

### 2.7. Anethole Synergy with Cisplatin in Inhibition of Many Cancers Signaling Pathways

**Figure 8:**
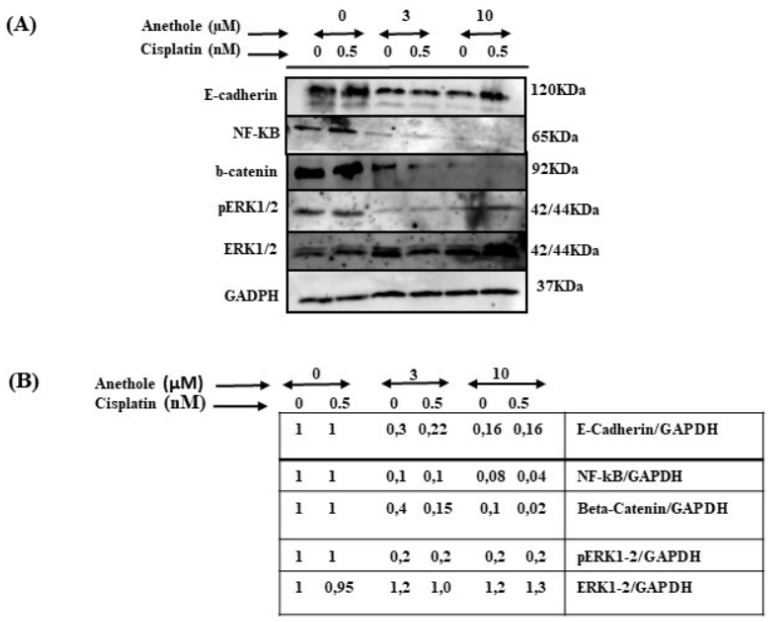
Effect of the combination of anethole and cisplatin on inhibition of many cancer signaling pathways. (**A**) The synergistic effect of anethole with cisplatin promotes the further inhibition of epithelial-to-mesenchymal transition genes and inhibits many cancer signaling pathways such as ERK1/2, beta-catenin, and NF-kB activation. (**B**) The quantitation analysis of the levels of proteins (*n* = 3). Some ratios are indicated after the scanning procedure and band intensity quantification using the NIH software program.

To investigate the impact of combined cisplatin and anethole in inhibiting the many pathways involved in cancer progression, we carried out an assessment through Western blot analysis by using one concentration of cisplatin (0.5 nm around IC_50_) with or without two concentrations of anethole (3 and 10 µM). As shown in Figure 8, the combination of 0.5 nM of cisplatin with 3 or 10 µM of anethole had a strong inhibitory effect on the ERK1/2 phosphorylation (activation) but not on the expression of total ERK1/2. A similar effect was observed for NF-κB (p62) activation. In addition, p62 expression decreased in response to a combination of 0.5 nM of cisplatin with 3 µM of anethole and was significantly more inhibited when we combined 0.5 nM of cisplatin with 10 of anethole µM. Similar data were observed for beta-catenin (Figure 8). These results show that a combination of cisplatin and anethole may inhibit the NFκβ and MAPK downstream signaling.

## 3. Discussion

The present study investigated the synergistic effect of anethole with cisplatin against oral cancer cell proliferation, showing that the combined treatment of cisplatin and anethole promotes and potentiates the cisplatin-induced inhibition of cell proliferation.

The inhibitory effect of anethole and cisplatin on oral cancer cells was concentration dependent when used alone. However, the combination of cisplatin and anethole showed a significant synergistic anticancer effect, which is the most noteworthy finding from our results (CI < 1). The IC_50_ of anethole single exposure, which was 10 µM and 0.6 µM for cisplatin alone, decreased significantly when the drugs were combined. When combined, the IC_50_ was reduced by 2.4-fold for the cisplatin combination with 3 µM of anethole, and 125 times with 10 µM of anethole. Similarly, a synergistic effect was observed for cell cytotoxicity and colony formation when we combined cisplatin with anethole. These data support the hypothesis that anethole potentiates the cisplatin effect and could be a potential complementary chemotherapeutic by reducing the concentration of cisplatin, thus, minimizing its side effects while maintaining the desired therapeutic response. To our knowledge, it is the first study to investigate the synergistic effect of anethole in association with cisplatin on oral cancer. Nonetheless, recent studies have reported an anticancer effect of anethole alone in inhibiting the proliferation of human prostate cancer cells [24]. It has also been reported to have an antitumor effect on breast cancer cell lines by inducing apoptosis and suppressing cell survival [25]. It was reported that many chemo-preventive phytochemicals having antitumor properties can overcome the side effects of chemotherapy resistance and its non-specific cell toxicity. These phytochemicals exert their effects by modulating many cellular proteins involved in several pathways involved in tumorigenesis. In the study by Meher et al. (2012), the highest synergy in the effect of anethole and curcumin with platinum-based drugs on epithelial ovarian cancers was observed when both compounds (platinum with phytochemicals) were added at the same time [26]. Other researchers have investigated the synergistic effect of cisplatin with other natural products such as berberine [27,28]. A study conducted by Yang Wang et al. in 2018, on the synergistic effect of capsaicin with cisplatin on osteosarcoma, showed that the combination of the two drugs significantly inhibited the proliferation of osteosarcoma cells compared to individual drugs [29]. Similarly, according to another study by Ho et al. on the synergistic anticancer effect of triptolide and cisplatin on cisplatin-resistant bladder cancer cells, they showed that treatment with both agents significantly inhibits proliferation [30]. This synergy in the inhibition of proliferation was explained by the ability of these two drugs to block the cell cycle. The antitumor activity of cisplatin was reported to be closely linked to its ability to DNA adduct formation and cell-cycle arrest after cisplatin treatment was reported in many cancer cells [31]. It was reported that, depending on cisplatin concentration and the time of exposure to cisplatin, many cells recover to a normal cell cycle or undergo cell death [32,33]. We strongly believe that the development of strategies aimed at slowing down cell cycle progression and enhancing cell death will open the prospect of increasing the chemosensitivity of cancer by combining these natural molecules known as interactive agents with conventional chemotherapies.

Cancer is defined as the balance between proliferation and apoptosis events. Targeting apoptosis is considered a new therapeutic approach to killing cancer cells. The effectiveness of traditional drugs in inducing apoptosis reduces over time due to the development of chemoresistance. The resistance of cancer cells to cisplatin primarily arises from the inhibition of apoptosis. Therefore, it is imperative to identify a potent adjuvant that enhances the apoptotic effect of cisplatin-based chemotherapy. Our research reveals that anethole could serve as a promising contender for augmenting the cisplatin-induced apoptotic effect in oral cancer cells by stimulating caspase activation. Data from this study are consistent with our previous study where we showed that anethole induced anticancer effects by targeting three principal processes involved in tumorigenesis such as apoptosis, autophagy, and oxidative stress [21]. Targeting apoptotic caspases in cancer treatment has been documented in the literature. Targeting apoptosis in many cancer therapies is based on the activation of caspases. Recently a new therapeutic strategy was developed focusing specifically on the activation of the individual caspases using gene therapy approaches or by using molecules able to inhibit natural inhibitors of caspases [34]. In addition, many upstream regulators of caspases have been defined as oncogenes or tumor suppressors [35]. Combined platinum drugs with phytochemicals have shown their beneficial effect on several types of cancer. Ho et al. demonstrated that triptolide and cisplatin co-treatment has led to a synergistic increase in apoptosis expression compared to cells in bladder cancer patients treated with triptolide or cisplatin alone [30].

Furthermore, oxidative stress is the result of increased levels of ROS, which is an important factor that aids cancer development and progression [36]. Our findings showed that the combination of anethole with cisplatin, on one hand, increases intracellular ROS levels and on the other hand, increases the mitochondrial oxidative stress, which might trigger apoptosis or autophagy of the oral cancer cells. These results are consistent with that of Aggarwal et al. who showed that a high induction of ROS further stresses cancer cells and induces apoptosis and autophagy [37]. Moreover, the researchers have largely noticed that there are cell-specific circumstances regarding the effectiveness of ROS manipulation strategies. This means that some cancer cells tend to stop growth or die by being exposed to ROS, while others do so by eliminating or decreasing ROS. The researchers explain this contradiction, that the difference lies in the magnitude of the level of ROS generated which determines whether the pro-cancer or anticancer signaling is activated [38]. Cisplatin treatment is known to induce mitochondrial ROS and thereby cause reduced energy production due to dysfunctional mitochondria [39]. However, one of the main functions of mitochondria is its ability to produce energy by oxidative phosphorylation, one of the key endogenous sources of ROS. As reported in our previous study, anethole is considered as having antitumor capabilities due to its ability to induce oxidative stress in oral cancer cells [21]. The synergistic effect of anethole with other natural molecules has also been reported on cancer cell proliferation, and cell cycle arrest and promotes ROS-mediated apoptosis [40].

Metastasis is directly associated with the induction of EMT destruction, MAPK, and Wnt pathways [41]. In our study, we demonstrated that anethole potentiates the anti-metastatic effect of cisplatin through blocking the migration/invasion abilities of cells and possibly suppressing the EMT likely due to the induction of epithelial markers the E-cadherin and vimentin. Transgenic mouse models have provided clear documentation of the direct involvement of EMT in tumor progression and metastasis. This is evident through the deletion of the E-cadherin gene which has led to the transition from a well-differentiated adenoma to invasive carcinoma in various cancer models. [1,2]. Numerous epithelial tumor cells have reported an increase in the invasion and adaptation of fibroblasts due to the downregulation of E-cadherin expression [3].

Various signaling pathways such as MAPk, WNT, and NOTCH have been involved in EMT regulation with the same physiological conditions. The frequent activation of major survival pathways such as MAPK, NF-kB, and beta-catenin has been reported to contribute to the development and progression of tumor growth. Targeting these signaling pathways that are upregulated in various types of cancers is well documented. The ERK1/2 pathway is altered in many human cancers and MEK inhibitors were the first drugs developed. Unfortunately, these MEK inhibitors did not perform as expected in the clinic despite their high potency and selectivity [42]. Cisplatin exerts DNA damage through p53 activation and MAPK inhibition leading to the inhibition of pro-survival proteins and consequent caspase activation and apoptosis [43]. Anethole also induces its antitumor activity by targeting ERK1/2, NF-kB, and beta-catenin pathways [21]. Anethole was reported to block both early and late response transduction by TNF via NF-kappaB, AP-1, JNK, MAPKK, and apoptosis [17].

## 4. Materials and Methods

### 4.1. Cells and Cell Culture Conditions

Ca9-22 cell lines (RIKEN BioResource Research Center, Tsukuba, Japan) were cultured in RPMI-1640 medium (Gibco; Thermo Fisher Scientific, Burlington, ON, Canada) supplemented with L-glutamine, 5% fetal bovine serum (FBS), and Penicillin/Streptomycin (Sigma-Aldrich, Oakville, ON, Canada). Cells were cultured at 37 °C in a humidified incubator with 5% CO_2_ atmosphere conditions.

### 4.2. Drugs

The starting point of the experiment involved diluting anethole obtained from Sig-ma-Aldrich Oakville, ON, Canada with methanol, resulting in a stock solution with a concentration of 3 mM. This stock solution was then utilized to create different anethole concentrations (0.3, 3, 10, and 30 μM). Cisplatin, purchased from the same source (Sigma Aldrich, Oakville, ON, Canada), was also utilized at various concentrations (0.1, 0.5, 0.8, and 1 μM) during the study.

### 4.3. MTT Assay

As described by Semlali et al, cancer gingival cells were exposed to varying concentrations of cisplatin and anethole, or left untreated, and cell growth was assessed using an MTT assay. The experiment was repeated five times to ensure accuracy [21]. Data from the MTT assay were used to perform a combination index (CI) analysis using CompuSyn software. CI values were used to determine the type of effect present, with values indicating a synergistic effect when less than 1, an antagonistic effect when greater than 1, and an additive effect when equal to 1.

### 4.4. Nuclear Staining by Hoechst Assay

Cells (1 × 10^5^ cells) were cultured per well of a 12-well plate and the cells were exposed to various concentrations of cisplatin (0.1, 0.5, 0.8, and 1 μM) with or without anethole (3 and 10 μM) for 24 h. After incubation, the cells were washed with PBS, and fixed with the fixation solution (75% methanol + 25% acid acetic) for 2 min, then washed and re-fixed with the same solution for 5 min. Afterward, the cells were washed three times with PBS and incubated for 15 min with Hoechst 33342. Finally, to eliminate the marker, cells were washed three times with PBS, and staining was analyzed using a fluorescent microscope. The experiment was repeated three times.

### 4.5. LDH Activity

As we have previously described [21], Ca9-22 cells were seeded at the same exposure and culture conditions as above. The culture supernatants were collected after exposing cells to anethole and cisplatin for 24 h. The LDH activity was measured using an LDH kit (Sigma-Aldrich, Oakville, ON, Canada). Triton was used as a positive control (100% of toxicity). The experiment was repeated four times.

### 4.6. Colony Formation Assay by Crystal Violet Staining

The in vitro clonogenic assay was used to measure colony formation. To this end, cells were seeded in 18 Petri dishes at a density of 10^4^ cells per dish and incubated overnight at 37 °C to ensure adherence. The following day, the cells were treated with cisplatin alone (at concentrations of 0.1, 0.5, 0.8, and 1 μM), anethole alone (at concentrations of 3 and 10 μM), or a combination of both, and then incubated for two weeks. After the incubation period, the cells were washed twice with PBS, fixed with cold methanol for 10 min at 4 °C, and stained with crystal violet dye for 10 min at room temperature. The cells were subsequently washed with deionized water and allowed to dry overnight before the number of different clones in each condition was counted. This experiment was repeated three times.

### 4.7. Wound Healing Assay

Cells (10^5^) were seeded in 12-well cell culture plates and exposed to different concentrations of cisplatin (0.1, 0.5, and 0.8 μM) with or without anethole (3 and 10 μM) for 24 h. Then a sterile 20–200 μL pipette tip was held vertically to scratch across each well. The image was acquired immediately and after 6 h. The pictures of each culture cell were performed by camera and analysis of the open wound area was measured with the calculation of wound area (percentage) before and after 24h of stimulation with drugs. The experience was repeated three times

### 4.8. Cell Apoptosis Assay by Annexin V-FITC and Propidium Iodide

The Ca9-22 cell cultures were subjected to various concentrations of the drugs being studied, either alone or in combination with an adequate concentration of anethole and cisplatin, or were left untreated for 24 h in a 5% CO_2_ humid atmosphere at 37 °C. Afterward, the cells were detached using 0.05% trypsin, washed with PBS, and then assessed for apoptotic cells using the annexin V-FITC/propidium iodide kit from BD Bioscience (Mississauga, ON, Canada). The cell suspensions were stained with 5 μL of annexin and 5 μL of propidium iodide and then analyzed for the percentage of live cells, apoptotic cells, and necrotic cells using flow cytometry (BD Accuri™ C6; BD Bioscience, Mississauga, ON, Canada). This experiment was repeated four times.

### 4.9. Cell Autophagy Assay

ICT’s autophagy assay (Red from ImmunoChemistry Technologies, Burlington, ON, Canada) was used as described by the manufacturer on cells treated with anethole alone (0.3 and 10 μM), cisplatin alone (0.1, 0.5, 0.8 and 1 μM), and their combination for 24 h. Stained cells were analyzed by flow cytometry using a green/yellow laser. The experiment was repeated four times.

### 4.10. Determination of ROS Levels

Oxidative stress was assessed by flow cytometry assessing the generation of reactive oxygen species (ROS) on either treated or untreated cells (ImmunoChemistry Technologies, Burlington, ON, Canada). Total ROS Green from ImmunoChemistry Technologies (Burlington, ON, Canada) was reconstituted and used as recommended by the manufacturer. ROS generation was detected by flow cytometry. The intensity of fluorescence was analyzed at 488 nm by BD Accuri C6 Flow Cytometry system (BD Bioscience). The experiment was repeated three times.

### 4.11. Determination of MitoSOX Levels

Mitochondrial superoxide interacting dye (MitoSOX Red; Molecular Probes, Invitrogen, Burlington, ON, Canada) generates fluorescence products, which were detectable by flow cytometry. Briefly, cells were treated with different concentrations of anethole (3 and 10 μM) and cisplatin (0.1, 0.5, 0.8, and 1 μM). Secondly, total MitoSOX™ Red was reconstituted with DMSO then diluted in the PBS, added to each sample (106 cells/mL), and incubated in the dark for 1 h at 37 °C. After incubation, the intensity of fluorescence was analyzed at 510 nm by BD Accuri C6 Flow Cytometry system (BD Bioscience), and the percentage of positive cells was calculated. The experiment was repeated three times.

### 4.12. ΔΨm (Mitochondrial Membrane Potential) Assay

To evaluate cell health, we investigated the changes in mitochondrial membrane potential (MMP) by using a Mito Probe TMDiOC2(3) assay kit from Thermoficher, as described by our previous work [44]. Ca9-22 cells were treated with each concentration of each drug or by a combination of cisplatin and anethole for 24 h. After washing with PBS, the cells were loaded with 5 µL of 106 µM of DiOC2(3) for 30 min at 37 °C in the dark before performing flow cytometry analysis. The experience was repeated three times.

### 4.13. Western Blot and Antibodies

Western blot was used as described in our previous work [24]. The antibodies used are as follows: NF-κB (sc-8008) and β-Catenin (sc-59737) were purchased from Santa Cruz Biotechnology (Santa Cruz, CA, USA); pERK1/2 (4370) and ERK1/2 (4695) were purchased from Cell Signaling Technology (Danvers, MA, USA), and GADPH was purchased from Sigma Aldrich. The secondary goat anti-mouse (554002) and anti-rabbit (554021) were from BD Pharmingen (Mississauga, ON, Canada). Each experiment was repeated three times.

### 4.14. Statistical Analysis

The experiments were repeated a minimum of three times, and the means ± SD of the experimental values were reported. The difference in statistical significance between the control (no anethole and cisplatin) and test (desired concentration of anethole and cisplatin) values was determined as a percentage. A one-way ANOVA was used to determine the statistical significance of the differences between the values, and a significant difference was defined as a *p*-value < 0.05.

## 5. Conclusions

We demonstrated that anethole in combination with cisplatin markedly inhibited oral cancer cell viability, and cell invasion capabilities, and increased cell apoptosis and autophagy, in vitro. In addition, the combination of anethole and cisplatin demonstrated a synergistic inhibitory effect on the viability of oral cancer cells. The combination treatment induced autophagy flux, leading to enhanced cell death, indicating that autophagy was a pro-survival process in cells. Our results suggested that the mechanism behind the effects of the combined treatment was initiated by acting on the generation of mitochondrial ROS. These data suggest that anethole could be a powerful new chemotherapeutic agent against gingival squamous cell carcinomas by modulating proliferation, cell death, and different signaling pathways. However, further studies are needed to better understand the molecular mechanisms behind the effects of this synergy and further investigate the signaling pathways involved in different oral cancer cells.

## Data Availability

Data is contained within the article.
